# Employing Participatory Citizen Science Methods to Promote Age-Friendly Environments Worldwide

**DOI:** 10.3390/ijerph17051541

**Published:** 2020-02-27

**Authors:** Abby C. King, Diane K. King, Ann Banchoff, Smadar Solomonov, Ofir Ben Natan, Jenna Hua, Paul Gardiner, Lisa Goldman Rosas, Patricia Rodriguez Espinosa, Sandra J. Winter, Jylana Sheats, Deborah Salvo, Nicolas Aguilar-Farias, Afroditi Stathi, Adriano Akira Hino, Michelle M. Porter, On behalf of the Our Voice Global Citizen Science Research Network

**Affiliations:** 1 Department of Epidemiology and Population Health, Stanford University School of Medicine, Stanford, CA 94305, USA; 2 Stanford Prevention Research Center, Department of Medicine, Stanford University School of Medicine, Stanford, CA 94305, USA; banchoff@stanford.edu (A.B.); jennahua3@gmail.com (J.H.); lgrosas@stanford.edu (L.G.R.); prespinosa@stanford.edu (P.R.E.); sjwinter@stanford.edu (S.J.W.); jsheats@tulane.edu (J.S.); dsalvo@wustl.edu (D.S.); 3 Center for Behavioral Health Research and Services, Institute of Social and Economic Research, University of Alaska Anchorage, Anchorage, AK 99508, USA; dkking@alaska.edu; 4 JDC Eshel, Jerusalem 91034, Israel; smadarso@jdc.org (S.S.); ofirb@jdc.org (O.B.N.); 5 Faculty of Medicine, The University of Queensland, Brisbane QLD 4072, Australia; p.gardiner@uq.edu.au; 6 Department of Physical Education, Sports and Recreation, Universidad de La Frontera, Temuco 4780000, Chile; nicolas.aguilar@ufrontera.cl; 7 School of Sport, Exercise and Rehabilitation Sciences, University of Birmingham, Edgbaston, Birmingham B15 2TT, UK; A.Stathi@bham.ac.uk; 8 Postgraduate Program in Health Technology (PPGTS), Polytechnic School, Pontifícia Universidade Católica do Paraná (PUCPR), Curitiba (PR) 80215-901, Brazil; akira.hino@pucpr.br; 9 Centre on Aging, and Faculty of Kinesiology and Recreation Management, University of Manitoba, Winnipeg, Manitoba, MB R3T 2N2, Canada; michelle.porter@umanitoba.ca

**Keywords:** citizen science, participatory research, older adults, aging, age-friendly environments, WHO, health promotion, health equity, digital health, built environment

## Abstract

The trajectory of aging is profoundly impacted by the physical and social environmental contexts in which we live. While “top–down” policy activities can have potentially wide impacts on such contexts, they often take time, resources, and political will, and therefore can be less accessible to underserved communities. This article describes a “bottom–up”, resident-engaged method to advance local environmental and policy change, called *Our Voice,* that can complement policy-level strategies for improving the health, function, and well-being of older adults. Using the World Health Organization’s age-friendly cities global strategy, we describe the *Our Voice* citizen science program of research that has specifically targeted older adults as environmental change agents to improve their own health and well-being as well as that of their communities. Results from 14 *Our Voice* studies that have occurred across five continents demonstrate that older adults can learn to use mobile technology to systematically capture and collectively analyze their own data. They can then successfully build consensus around high-priority issues that can be realistically changed and work effectively with local stakeholders to enact meaningful environmental and policy changes that can help to promote healthy aging. The article ends with recommended next steps for growing the resident-engaged citizen science field to advance the health and welfare of all older adults.

## 1. Introduction

“Never doubt that a small group of thoughtful, committed citizens can change the world; indeed, it’s the only thing that ever has.” Margaret Mead

Over the past two centuries, improvements in an array of social, environmental, and biological factors, including sanitation, housing, education, and medical care, have led to overall longevity increases worldwide [1]. It is estimated that by 2050, 1 in 5 people will be 60 years of age or older [2]. Yet, for a growing number of adults today, longevity increases have not been accompanied by better health compared to prior generations [3], and this is particularly true among disadvantaged populations [4].

In light of this alarming trend, the World Health Organization has recommended a global strategy whereby all populations, regardless of geographic region, living conditions, or economic circumstances, can benefit from evidence-based activities aimed at maximizing functional ability and health [2]. Among the key strategies described in this call for action is the development of age-friendly environments. This is due to increased recognition of the substantial impacts that local environments have on older adults’ continuing health, mobility, activities, well-being, and quality of life [5,6,7].

The central strategies identified in developing age-friendly environments include fostering older adults’ engagement and autonomy and facilitating multi-sectoral action [2]. While much has been written about the important role that older adults themselves can play in contributing to building an age-friendly community [8,9,10], this area can continue to be advanced through systematically deploying and testing purposeful and sustained resident engagement models in developing, evaluating, monitoring, and implementing changes to improve the age-friendliness of their environments [11,12]. Notably, in what has been referred to as the “paradox of neighborhood participation”, older adults typically spend a large amount of time in their neighborhoods but are often among the last to be included in local decision-making activities [8,13]. Involvement in such activities is strongly recommended by experts in the field as well as the WHO Global Age-Friendly Cities movement [8,14].

Participatory research—which can be defined as community participation and involvement aimed at promoting greater public transparency and data accessibility in a research project’s decisions and processes [15]—has been expanding in a variety of fields in pursuit of environment-based knowledge and improvements. In addition to the public and population health areas, other fields in which participatory research activities have been well represented are public participation Geographical Information Systems (GIS) and volunteered geographic information research [16,17,18,19], and regional and environmental planning and management [20,21].

Involving older adults in these processes can enhance their perceptions of autonomy, empowerment, and collective agency as they witness how their own actions can lead to tangible improvements to their local environments [22]. For instance, significant pre–post increases in neighborhood cohesion (e.g., “This is a close-knit neighborhood”) and community engagement and collective agency (e.g., “By working together with others in my community, I can influence decisions that affect my community”) have been reported among low-income Latino older adults participating in a citizen science project aimed at barriers to active living [23,24]. In addition, behavioral evidence of residents’ enhanced empowerment and agency beliefs has occurred when older adults trained in community participatory action processes around one local issue (e.g., inadequate food access) were observed subsequently to independently generalize their advocacy activities to other local issues (e.g., advocating for increased funding for affordable senior housing with state policy makers; joining forces with a local school in engaging with the city’s planning and transportation department to improve intersection safety [i.e., flashing signal lights and crosswalks] for pedestrians and cyclists) [25].

It should be noted that participatory research models vary regarding the extent of participation expected from older adults. For example, in the Quebec 3 step program, older residents were consulted primarily through focus groups, as were appropriate service providers [26]. This information was then used by a community steering committee of stakeholders, including representatives from relevant organizations and institutions, who recommended actions to be taken. Older adults served as consultants for the program but did not appear to participate in actual community-specific data generation, analysis, or interpretation, or participatory action planning. In contrast, as part of an age-friendly program in Manchester, project developers have actively engaged older adults as co-researchers to improve the quality of life of older residents [8]. The older adults have been directly involved in the leadership aspects of the initiative [27], with the project leading to concrete policy outcomes to foster age-friendly communities as well as local transit improvements (i.e., restoration of a bus service in one of the neighborhoods being studied) [28].

The growing field of health-related citizen science represents one means of engaging older adults directly in contextually relevant participatory research that can benefit not only their own health, but the health of their communities [29,30]. Citizen science, broadly defined as non-scientists participating in the research process to advance science [31], is a centuries-old tradition in some countries, such as the USA [32]. In the traditional citizen science context, “citizen” has been defined simply as an inhabitant of a particular town or city (without regard to legal status), and it is that definition that is employed in this article. Part of the family of approaches collectively referred to as participatory research, citizen science approaches often have brought systematic, scalable methods of resident-based data collection to the scientific endeavor.

The citizen science field is comprised of different levels of resident engagement that can be placed broadly into the following three categories [22]: (a) citizen science “for the people”, which typically is limited to donations by residents of biological specimens or other forms of personal information to advance biomedical or other types of research; (b) citizen science “with the people”, which has been used extensively in the natural and biological sciences, including astronomy and ecology, and usually involves systematic data collection by residents around specific observable phenomena, with the data then sent to scientists or other groups to analyze and interpret (e.g., to municipal authorities, in the case of mobile apps that encourage residents to photograph local problems, such as potholes, which can be sent to a specific website); and (c) citizen science “by the people”, which is viewed as a partnership between residents, researchers, and relevant community organizations. In this latter citizen science category, also referred to as participatory action research, residents typically contribute to study objectives and/or questions of interest, data collection and interpretation, and development of relevant actions based on the results [22].

One example of the “by the people” citizen science approach to participatory action research is called *Our Voice* (24). *Our Voice* employs easy-to-use mobile technology as a gateway to engaging and activating socioeconomically diverse groups of older adults in advancing the WHO’s age-friendly communities initiative [24]. To date, the *Our Voice* methods have been used in over 30 participatory research projects around the globe, with fourteen of these specifically involving older adults. A central question underlying this research concerns whether older residents, irrespective of locale or culture, can participate sufficiently in the *Our Voice* method to drive changes in local environments that support healthy aging. The major aim of this article is to describe this citizen science approach and highlight results from *Our Voice* research projects around the world that have tackled specific challenges relating to the built, social and services environments—domains that are deemed critical to promoting age-friendly and health enhancing communities identified in the WHO aging and health reports [2,33,34]. The article ends with recommendations for next steps in using “by the people” citizen science approaches, such as *Our Voice*, to advance the healthy aging participatory research field.

## 2. General Methods and Materials for the *Our Voice* Citizen Science Engagement Model

### 2.1. Overview

The major goal of the *Our Voice* citizen science model is to empower residents, regardless of geography, age, or socioeconomic and cultural backgrounds, to activate health-promoting changes in their local neighborhoods and communities in collaboration with relevant community organizations and academic partners [22,24]. While the term “empowerment” has a number of different definitions that are relevant to this work, Rappaport’s definition (1984) can be generally employed, i.e., “the process by which people, organizations, and communities gain mastery over their lives” [35]. While varied surveys have been used to measure empowerment and similar constructs, frequently used questions have been drawn from the social capital module of the General Household Survey 2000 [36].

*Our Voice* sits within a socioecological framework of impacts, spanning person- to policy-level outcomes [37,38], and is informed by behavioral and social action theories of change (e.g., social cognitive theory [39], self-determination theory [40], social action model of community engagement [41,42]), and implementation science theory aimed at maximal scalability and sustainability [43]. It represents a form of trans-directional ecological model in which reciprocal person by environment interactions are specifically targeted as a means of creating impacts at multiple levels (i.e., individual, built and social environments, policy) [44]. The goal of such models is to actively cross levels of impact through using agents at one level (individual community members) to actuate changes at higher levels of impact (environment, policy) [44].

The *Our Voice* model combines key strengths of traditional forms of citizen science and community-based participatory research (CBPR) methods. These include greater standardization of resident data collection methods—a hallmark of citizen science—than what is often found in CBPR applications, and more complete involvement of residents in the full scientific endeavor relative to more frequently used citizen science methods [13,22]. (See *Our Voice* video overview in Supplementary Materials.)

**Figure 1:**
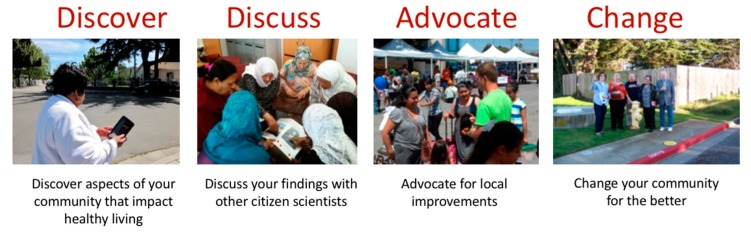
The 4 step *Our Voice* citizen science model [24]. © Stanford University. All rights reserved.

The 4 step *Our Voice* process is summarized in Figure 1. Prior to starting an *Our Voice* project, the facilitating organization (e.g., research team, community group, government agency) participates in remote, web-based development of project goals and an implementation plan. They then receive remote training on program methods and ongoing project support. The project team members next recruit residents as Citizen Scientists and orient them to the project and their role. Using a multi-lingual mobile app, called the Stanford Healthy Neighborhood Discovery Tool^TM^ [45] (described in more detail below), residents capture, through geo-coded photographs and audio- or text-based narratives and route mapping, features of their local environments that help or hinder a particular domain that can impact healthy living, for example, neighborhood walkability, food access, personal safety, feelings of support and respect, transport, or well-being [25,46,47,48,49,50,51,52]. In a standardized procedure that occurs across projects, the qualitative data collected using the Discovery Tool are automatically uploaded to a secure server where the different data elements (e.g., photos, narratives) are combined and then transmitted back to designated project personnel for distribution to participating residents. Then, in a facilitated group process, the residents share their data with other citizen scientists, categorize the data into relevant themes, interpret the data with respect to importance for the targeted issue under consideration, prioritize areas of concern, brainstorm feasible strategies and solutions for action, and identify local stakeholders, policy makers, and other potential allies with whom to discuss the issues further. Next, they meet with these local stakeholders to present their findings, discuss realistic solutions, and develop initial action steps for positive change in the identified areas. Typically, a total of two to three formal group meetings occur (the first with community member participants, then with the community member participants and relevant stakeholders) as part of the *Our Voice* process. This type of citizen science process has been found to be highly efficient and minimally burdensome, with suitable agreement/convergence around primary environmental barriers and enablers in a particular locale often achievable with as few as 8–10 residents [24,53]. While all projects employ the 4 step sequence summarized in the figure below, the model allows for customization of activities within each step commensurate with the contexts and constraints attendant with the particular population. All projects also are encouraged to include relevant multi-level measures, as described in a previously published *Our Voice* Network scientific article [44]. Final determinations concerning the most relevant assessment battery for the population being targeted rest with each project group.

As noted above, the *Our Voice* program begins with the Discovery Tool (DT), an easy-to-use mobile app that was developed originally for low-income older adults [45]. It has been used with residents ages 9 to over 90 years old to document features of their local neighborhoods or other environments that impact specific aspects of their health or well-being (e.g., physical activity, food access, personal safety, feelings of inclusiveness) [23,24,45,51]. The DT currently has been translated into ten languages. Language translation is readily accomplished, given that the design of the app uses universal symbols and graphics, with few written words. Data captured by the DT include geographical information systems (GPS) route tracking and geo-coded photos and audio or text narratives of local features, with visual ratings of each feature as either positive (green “smiley face”), negative (red “frown face”), or both (i.e., ability to record both). This spatial qualitative data method allows for the capture of residents’ experiences of their community in situ. Such data may better reflect environmental elements of particular importance to older adults relative to more frequently used questionnaires or interviews that typically rely on recall or more global assessments of walkability or safety [54]. Interestingly, some research suggests that assessments typically collected away from the outdoor spatial contexts of interest (e.g., surveys, interviews, focus groups) may lead to different results, particularly in older adults who often have developed unique environmental needs and lower memory/cognitive capacities [55]. Successful training in the use of the app typically takes about five minutes. Given that the primary focus of the *Our Voice* method is on enhancing local environments (i.e., places and spaces), residents are instructed to take photos of locations, not people (if faces or other identifiable data are inadvertently recorded, they can be deleted or blurred upon upload to the secure server). This also diminishes any ethical or practical issues that can accompany taking photos of individuals without their knowledge or consent. After collection, the data are uploaded onto a secure Stanford University server, where the photos, narratives, walk maps and user ratings are integrated into project reports. The project reports are then returned to the facilitating organization for participant distribution and discussion/analysis. The Discovery Tool secure data repository goes through annual approval by the Stanford University Institutional Review Board (IRB) for the protection of human subjects (IRB protocol #40379). Collaborating research organizations also obtain human subjects/ethics approval from their respective academic institutions. Non-academic partners collaborate with Stanford under Stanford’s IRB protocol #45330.

The *Our Voice* citizen science model has been applied or is currently being tested in over 20 countries across six continents in response to a range of local issues that can impact health. A major goal of *Our Voice* projects has been to engage underserved populations that typically have lacked a voice in decision making related to their local neighborhoods and communities. The promotion of health equity (i.e., everyone has a fair and just opportunity to live the healthiest life possible) [56] is a principal theme of this work. As noted earlier, *Our Voice* has been described as a “bottom–up” approach to environmental change that can complement or extend more traditional “top–down” policy-oriented approaches to change [24].

### 2.2. Characterizing Our Voice Project Initiatives Aimed at Built, Social and Community Service Environments

Using the WHO Age-Friendly Cities guide (13) and checklist (14), we reviewed the fourteen *Our Voice* projects conducted with older adult populations. We categorized the key barriers and action steps identified and local changes proposed and carried out within each of the three major domains and related eight topic areas promoted by the WHO as essential to support healthy aging. The key domains, based on the WHO Age-Friendly Cities guide, are the following: (a) built environment (e.g., outdoor spaces and buildings, transportation housing); (b) social environment (e.g., social participation, respect and social inclusion, civic participation); and community and health services (e.g., communication and information, community support and health services). These domains, along with the specific age-friendly topic areas they address, also drawn from the WHO Age-Friendly Cities guide, are summarized in Table 1. While many of the domains and topic areas overlap, the three key domains provide a useful rubric to highlight the potential of *Our Voice* methods to produce specific action steps and changes that are locally and internationally relevant from an age-friendly perspective. Because not all projects were conducted in cities, we have substituted “communities” for “cities” at appropriate places throughout the paper.

**Figure 2:**
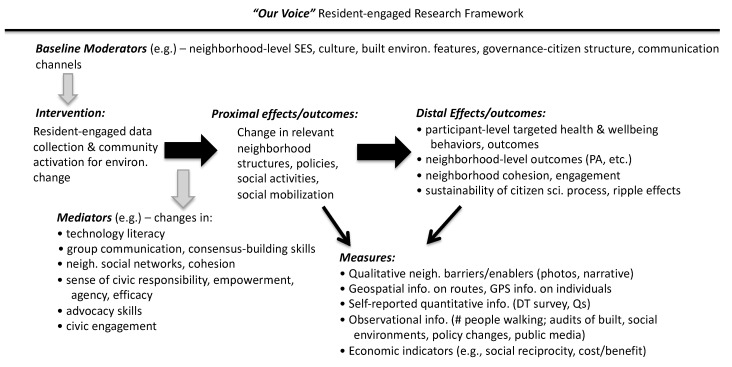
*Our Voice* research framework.

The *Our Voice* research framework is summarized in Figure 2 [24,38]. The framework posits that the *Our Voice* intervention program, through its impacts on an array of relevant mediators associated with the intervention, such as increased resident engagement, can lead to changes in proximal outcomes, including neighborhood structures, policies, and social activities, and, if sustained, more distal outcomes (e.g., individual, neighborhood, and community-level changes in health behaviors and outcomes; spill-over effects to other issues and problem areas that residents deem relevant). The intervention’s effects can also be moderated by local factors, such as neighborhood socioeconomic status, local governance structures, and built environment features.

Table 1, below, provides a brief overview and description of some of the varied *Our Voice* older adult projects that have been completed or are in process.

**Figure 3:**
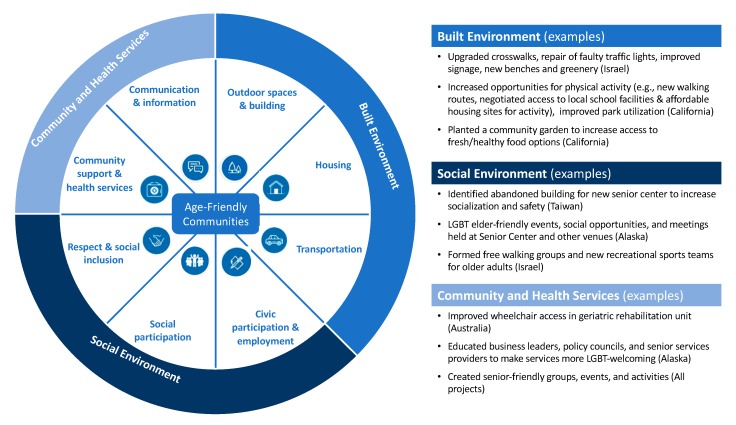
Topic areas underlying global age-friendly communities, adapted from the WHO [33].

The age-friendly domains and topic areas are shown in Figure 3 below, along with examples reflecting the range of outcomes identified from different *Our Voice* locales thus far.

## 3. Results

In this section, we present examples of how *Our Voice* has been used to address the three age-friendly community domains (i.e., built, social, or community and health services environments) and associated age-friendly topic areas in different geographic areas globally, including several previously unpublished citizen science studies. These examples are also intended to highlight how this community engagement model can be used across diverse locales and populations to facilitate scalable and sustainable local changes to promote healthy living. Consistent with the principles of participatory action research that emphasize involvement of older adult co-researchers in defining the problem and solutions [13], “success” related to environmental and policy changes targeted by the older adult citizen scientists in each project was defined based on whether the solutions implemented addressed identified problems and contributed to Age-Friendly communities, as defined by the WHO framework [33]. The environmental and policy changes occurring as part of the older adult citizen scientist efforts that are described in each project were verified via observation by the research and organizational partners facilitating each project.

Lessons for sustaining resident momentum during and beyond the project period are briefly summarized in a subsequent section.

### 3.1. Enhancing Built Environments to Promote Active Aging

Decades of research has demonstrated the relationship between the physical or “built” environments in which we live and activity engagement throughout the life course, including walking and recreation [57,58]. From an age-friendly community perspective, the design of outdoor spaces, buildings, and transportation are critically important for assuring their accessibility, safety, and attractiveness for older adults, who may face a range of mobility and sensory impairments [57,58]. Of specific importance is assuring that public areas are clean, green, and include outdoor seating; and that pedestrian walkways are free of obstructions, trip hazards, cyclists, cars, or other safety hazards. The following two projects illustrate the use of *Our Voice* methods to create age-friendly outdoor spaces for walking and other desirable recreational activities.

#### 3.1.1. Improving Neighborhood Walkability for Israeli Older Adults

To evaluate barriers and enablers of neighborhood walkability and walking routes among older Israeli adults, an initial study using the *Our Voice* Citizen Science method was [54,59] coordinated by JDC Eshel, the association for the planning and development of services for older adults and their families in Israel, in partnership with the University of Haifa (with university institutional review board approval). The study was conducted in neighborhoods in the city of Haifa that represented the socioeconomic diversity of the city [54]. The project team recruited 59 independently living adults ages 50 years and older who were equally distributed across the neighborhoods. Citizen science participants were recruited through mailed and posted flyers distributed throughout the neighborhoods as well as word-of-mouth among community members. Participants were successful in using the Discovery Tool app to capture >295 audiovisual pieces of data identifying relevant barriers to and enablers of local walking routes in their local neighborhoods [54]. Through subsequent facilitated group discussions (averaging two per neighborhood) and dialogue with local municipal decision makers, they also were able to successfully identify the safest routes to relevant destinations. Together they developed a senior-friendly “golden path” map and worked with the Mayor’s office and other organizations, including some local businesses, to initiate changes (e.g., improved aesthetics) to better support walking [59].

The successes from this initial evaluation led JDC Eshel to expand the use of *Our Voice* citizen science activities to 29 neighborhoods across nine other cities in Israel. The overall goal of the citizen science initiative is to improve seniors’ local environments in support of walking and related health-promoting activities. Thus far, 322 residents have engaged in citizen science activities, and over 1000 residents have participated in various healthy lifestyle activities following this project. The citizen science participants were ethnically and socioeconomically diverse, and in some cases, youth or young adults were invited to engage in the neighborhood citizen science process with the older adults (e.g., in Jerusalem, Tel Aviv). (See Table 2 for summary information on the first five cities that have completed their projects. The remaining cities are in the final phase of their projects.) While this citizen science initiative is ongoing, successes thus far have included upgrading of crosswalks; repair of traffic signs and extension of the length of time traffic lights remained green to allow for easier street crossings; planting of trees and greenery to enhance local aesthetics; addition of fences along roadways to direct pedestrians to safer places to cross; and installation of benches along routes to supermarkets and recreational clubs. In addition, formation of free walking groups for seniors and development of a recreational sports team for older adults at local community centers addressed an identified need to improve social support for engagement in physical activity. A key to the project’s success was the active involvement of diverse community stakeholders and decision makers (e.g., nurses, social workers, municipal welfare departments, city government officials, directors and personnel from community “golden age” clubs for older adults). Participants also reported increased feelings of empowerment, collective efficacy, and neighborhood connectedness across the participating citizen science groups, reflected in the following participant quotes: “I felt I was an influencer—people listened”; “Participating in the project made me look differently at the ways I go [to get places]; I’m more careful today”; and “I love my neighborhood, I was born in it and I also want to grow old in it. It is important to me that it be safe for me, that I will not fall, and will be able to walk safely. I want to keep taking photos even when the project ends.”

#### 3.1.2. Creating Convenient Multi-Generational Physical Activity and Recreation Opportunities in San Jose, CA

In a multi-generational project that included 50 adults and youth in the Mayfair area of San Jose, CA, the community-based SOMOS Mayfair non-profit organization partnered with Stanford researchers and the Santa Clara County Public Health Department to identify barriers and develop solutions to promote active and safe living in this ethnically diverse, historically underrepresented area (e.g., 79% of residents speak a language other than English at home). The data that residents collected using the Discovery Tool and around which consensus was subsequently built were presented to the Mayor of the city of San Jose and City Council members. Among the successes that occurred from this project were the development of a memorandum of understanding with the local school district to allow residents to access a local soccer field; designation of scholarships for enrichment programs at the local community center; development of “scavenger hunt cards” to promote use of a local park; creation of walking routes aligned with historical aspects of their neighborhood and resident-led walking groups; and physical activity programming in conjunction with National Night Out activities and the local Viva Parks program. These activities together increased opportunities for physical activity and improved park utilization as observed and documented by the System for Observing Play and Recreation in Communities (SOPARC) [60]. The results of this project show how resident-centered data-driven methods can provide a means through which historically underserved residents of all ages can work effectively with local decision makers and researchers to address long-standing social and environmental disparities that can impact health in their locales. As summarized by SOMOS Mayfair’s Executive Director (Camille Llanes-Fontanilla, MPA): “Through the *Our Voice* process this partnership has created a space for families to envision a neighborhood where residents of all ages can live and thrive”.

#### 3.1.3. Other Projects Aimed at Enhancing Built Environments to Promote Age-Friendly Communities

In addition to the above projects, examples from several other *Our Voice* projects that have been aimed at enhancing local environments to improve access to a variety of desirable physical and recreational opportunities are summarized in Table 1. Briefly, changes accomplished by these projects include creating a community garden adjacent to senior housing in a low-income northern California community [46,61]; reducing impediments to walking and addressing waste management in a low-income Latino neighborhood in the San Francisco Bay area, CA [23]; and developing local solutions to control stray and roaming dogs in Cuernavaca, Mexico [48]. Other projects in progress, some of which are described in Table 1, include improving the accessibility and navigability of the university campus in Manitoba, Canada; increasing the age- and activity-friendliness of diverse communities in West Midlands, South West and South East England; promoting environments that support healthy aging in Temuco, Chile and Curitiba, Brazil; and improving neighborhood walkability around senior affordable housing sites in San Mateo and Santa Clara Counties, California. In addition, *Our Voice* citizen science projects are being pursued in these latter counties to foster intergenerational and multi-cultural sharing around transportation and transit equity, and to enhance age-friendly cities, including safe routes for seniors programming. Innovative citizen science work also has been accomplished by Tuckett et al. in Brisbane, Australia, where older residents have contributed to solutions to enhance local walking infrastructure, including the repair and improvement of footpaths, and local park use, including municipal approval for installation of new toilets and exercise equipment [53]. Finally, projects are underway that highlight how age-friendly city-wide coalitions that include local municipal agencies and senior-focused non-governmental organizations (e.g., the American Association of Retired Persons [AARP]) can partner with academic researchers to increase accessibility for all, including older adults, in popular city districts. One such project, occurring in Seattle/King County, Washington state’s historic Pike Place Market, has generated 35 Discovery Tool walks, a total of 425 photos and 423 audio narratives, and has resulted thus far in improvements in signage and accessibility to the Market’s outdoor garden area.

### 3.2. Enhancing Social Environments to Promote Social Participation, Safety, Respect, and Inclusion

The *Our Voice* projects described above have focused principally on features of physical environments that impact lifestyle behaviors and similar factors of importance to healthy aging. Yet, local community features also can strongly impact social environments, including features that influence perceived safety and satisfaction with local services, and those that foster participation, respect and social inclusion [62]. These social determinants of health are equally important to older adults’ daily well-being and quality of life [63].

#### 3.2.1. Creating Safe, Senior-Friendly Social Spaces in Cijin, Taiwan

Taiwan’s population is aging at a rate more than twice that of Europe and the U.S. [64]. Yet, it is currently unclear how best to create age-friendly environments that meet the needs of the older adult population. The *Our Voice* Discovery Tool and citizen science process was used to capture older adults’ perspectives about their local environments in a contextually valid manner [65] (institutional review board approval from Kaohsiung Medical University, #kmuh/irb/af/08E-02). Fifteen older adults (mean age = 70.3 years [SD = 9.9], 33% women) living in Cijin, a small community in southern Taiwan, used the Discovery Tool during walks in their village to capture barriers to and enablers of healthy aging. A total of 78 photos and audio-narratives were collected. Issues that were identified by the citizen scientists included lack of public spaces for older adults to gather and socialize, abandoned buildings, a dysfunctional sewer system, cracked and broken sidewalks, and personal safety issues related to motorbikes and other factors. During one facilitated resident meeting, residents prioritized abandoned buildings and personal safety as high-priority issues that they would like to see addressed. Three weeks after this meeting, residents met with local village officials to share results and brainstorm potential solutions. An abandoned building was identified to turn into a community center where older adults could safely gather and socialize. However, turnover of project facilitators (which included students from a nearby university) contributed to a loss of momentum, and consequently the early gains that had been made in support of the building remodeling process stalled. In addition, there was a lack of clarity around which municipal entity—the university hospital that owned the building or the city of Cijin—was responsible for the remodeling costs. As a result, the remodeling of the building was not completed. Thus, while older residents were successful in using the Discovery Tool and *Our Voice* process to identify local issues impacting healthy aging and develop, with stakeholders, potentially feasible solutions, this study also underscored the importance of continuity among project facilitators, and the need to clearly identify “implementers” with the authority, interest and resources required to accomplish the requested change.

#### 3.2.2. Promoting Community-Wide Respect and Inclusion for LGBT Elders in Anchorage, Alaska

Lesbian, gay, bisexual and transgender (LGBT) elders often experience social stigma, loneliness, social isolation, and discrimination that can result in health disparities [66]. A pilot project conducted in Anchorage, Alaska, with LGBT elders represents the first project to assess the feasibility of using *Our Voice* citizen science methods focused specifically on promoting respectful, safe, and inclusive community environments [67]. In partnership with local branches of two U.S. national organizations supporting older adults, a convenience sample of eight LGBT Alaskan aging adults (mean age [SD] = 63.3 [6.7]; range = 53–71 years; 50% women) completed baseline and 6 month follow-up surveys about their health, perceptions of neighborhood social cohesion [68], loneliness [69], and access to LGBT-friendly services. Following baseline, citizen scientists completed a walk- (seven participants) or drive-about (one participant) using the DT to document, through 66 geo-coded photos and 65 recorded audio narratives, environmental features that enabled or hindered safe and healthy aging. A “drive-about” was used when a participant had a mobility impairment that limited his/her ability to engage in sustained walking. The car was driven by a volunteer, while the participant directed the driver, took photos, and recorded why each photo was taken. After completing the DT assessments, citizen scientists, advisors from the two national organizations (SAGE and the American Association of Retired Persons [AARP]), and LGBT advocates came together during four facilitated meetings to analyze and prioritize the DT data and develop potential solutions. To guide deductive theme generation, the group used the *WHO Checklist of Essential Features of Age-friendly Cities* [34] as a starting point. Participants subsequently met twice more to finalize key issues, brainstorm and prioritize possible solutions, and plan next steps.

The findings suggested that personal safety, respect, inclusion, social participation, and connectedness were hindered by lack of safe public transportation and information about LGBT-friendly places. For example, people loitering in front of public buildings, such as the public library, and youth disrespecting older adults were concerns for all participants but were noted as especially threatening for transgender elders. All described a heightened sense of vigilance when out in public or in social settings, such as senior centers, where they felt conscious of or wary about disclosing their sexual orientation or gender identity.

Of particular interest, participants reported meaningful increases in perceived social cohesion and decreases in loneliness after participating in the project for six months (effect size *d* = 0.42 and 1.03, respectively). For example, on the loneliness scale, the item with the most improvement was “I often feel rejected,” which went from 100% indicating that they felt rejected at least some of the time or often at baseline, to 25% at follow-up. Similarly, the item “There are enough people I feel close to” improved from half of respondents answering affirmatively, to 75% of respondents indicating that they agreed with that statement at 6 months. Follow-up assessments also indicated an increased perception that there are not enough psychological support groups for LGBT people and that community fear or dislike of LGBT people is a problem in Anchorage. A possible explanation was that listening to other participants’ experiences during the group meetings heightened individual awareness of issues that may or may not have matched their own experiences. With respect to social participation, citizen scientists described a general lack of information about low or no-cost LGBT-friendly events that could be attended alone or with a companion.

Feasible solutions that were identified through the citizen science engagement process included sharing their *Our Voice* discoveries through presentations to service providers, policy makers and business leaders, and creation of opportunities to connect with others by offering community partner-facilitated ridesharing to SAGE Alaska and AARP Alaska-sponsored events. At the end of the pilot study, citizen scientists expressed interest in sustaining their momentum by developing partnerships with businesses and community groups with a shared interest in creating a safe and inclusive city. Citizen scientists felt they could play a key role in helping to raise awareness of age-friendly needs and solutions to address inequities and, through SAGE Alaska, providing educational opportunities to senior centers, fitness clubs, and senior service agencies to help promote greater inclusiveness. The citizen scientists and LGBT advocates also expressed interest in broadening future efforts to engage LGBT youth in data collection and activities that can enhance social participation, respect, and inclusion across the lifespan. As of this writing, the citizen scientists have presented their findings to municipal, state and national audiences, including community partner board meetings, business leader breakfasts, the Anchorage senior center, and several scientific conferences [67]. Through SAGE Alaska, Identity, Inc. (a statewide advocacy organization for LGBT), and AARP Alaska, they have instituted ongoing social opportunities, including a weekly morning “coffee and conversation” event, held at a local café. They also are encouraging a more inclusive climate at the local senior center by using the facility for SAGE team meetings and special events. This exploratory study sets the stage for further, larger-scale investigations of this citizen science model as a potential method for improving inclusive social environments for all.

The above two projects demonstrate the importance, when assessing the age-friendliness of communities, of paying particular attention to environmental features and social barriers that may lead older adults to feel unwelcome or fearful [70]. Solutions that are generated should universally consider the needs of diverse older adults to diminish loneliness and isolation [62].

### 3.3. Increasing Access to an Age-Friendly Community and Health Services

An important, but understudied, age-friendly communities’ domain is one where the built and social environments collide, i.e., the health and social services sector [71]. The WHO emphasizes that community and health services, including clinics, hospitals, pharmacies, and social service settings, must be convenient and fully accessible for people with physical and cognitive disabilities [33]. Providers should be respectful and recognize the needs of diverse older adults, including language, culture, and relationships [71]. Communities should also assure that clear and accessible information about locally-relevant services is available and accessible to older adults, so they know what is locally available to support their ability to age well [33,71]. The following two examples emphasize the importance of built and social features to assure that older patients can not only navigate the physical settings where services are provided, but also can readily find out about trustworthy, welcoming services that exist within their community.

#### 3.3.1. Optimizing Comfort and Mobility in a Geriatric Medical Rehabilitation Setting

In the first *Our Voice* citizen science project occurring in a health care setting, ten patients (eight of whom used wheelchairs) used the Discovery Tool to assess features of a geriatric assessment and rehabilitation unit of a hospital in Brisbane, Australia, related to helping the rehabilitation process. The patients (eight men, two women) had a mean age of 56.7 [SD = 16.2] years. Human Subjects approval was received from the hospital’s human research ethics committee. The data collected by the citizen scientists using the Discovery Tool, which generated 49 photos and audio narratives and was discussed in two group sessions, indicated that a major factor impacting patients’ rehabilitation experience were environmental elements that were unfavorable for wheelchairs. Features that created barriers for wheelchair users included doors on cupboards and cabinets in bedrooms swinging outward to open, as opposed to sliding doors; shelves and hanging rails in cupboards that were difficult to reach; narrow doorways that were difficult to maneuver through for novice wheelchair users; basins and water dispensers that were difficult to access from a wheelchair; drab décor including curtains around beds that provided little privacy; and an inclined main entryway to the building that was challenging to use. Positive environmental features that were identified as enhancing the rehabilitation experience included the community garden and coffee shop on campus, as well as windows that provided views of the sky and some greenery for patients who could not leave the unit. In response to the citizen scientist data and information, the rehabilitation unit has initiated modifications, including moving a patient kitchenette and water fountain to more accessible locations; buying and hanging new curtains around beds to provide more privacy and brighten the feel of the unit; lowering paper towel dispensers in bedrooms; and rearranging furniture on the balcony to make it easier for patients in wheelchairs to navigate. Other initiatives, such as replacing furniture in the bedrooms, are being investigated.

Future directions relevant to improving the age-friendliness of community and health services domains include sharing data collected using *Our Voice* methods to inform providers about local environmental barriers that may impede adherence to treatment plans and prescriptions (e.g., difficulties accessing healthy foods, challenges engaging in regular walking, transportation barriers). One key feature will be providing patients with information about where they are allowed to be in a clinical setting. One of the barriers to patient mobility in hospital settings is that patients are often unclear as to where they can appropriately walk. Providing such information can open the door to additional productive interactions with patients that could not only improve built and social environments, but also enhance subsequent treatment adherence.

#### 3.3.2. Enhancing Communication and Information to Connect Older Adults to Community and Health Services

In addition to navigating physical environments in both community and health care settings, enhancing the communication channels used by service providers to reach older adults and, conversely, used by older adults to locate relevant, competent and quality services, is an important component of age-friendly communities. Assuring all older adults can access clear, accurate and up-to-date information about services, events, and opportunities of interest may improve access to a wide range of supports to meet their needs. An example of how this issue can be addressed was observed in the Anchorage, Alaska, *Our Voice* project described earlier, where LGBT participants attributed lack of information about LGBT-welcoming service providers, venues, and events as limiting their social and health-related activities. While residents felt that “lack of information” itself was challenging to photograph using the Discovery Tool, engagement in the environmental assessment heightened citizen scientists’ awareness of these less-concrete impediments to health.

### 3.4. User Experiences with the Discovery Tool App and Overall Our Voice Process

Across the projects included in this review, researchers and residents alike were generally pleased with the simple yet engaging functionality of the Discovery Tool mobile app—the entryway into the 4 step *Our Voice* process. While, with all forms of technology, the inevitable glitches occurred from time to time with app connectivity and similar functions, the issues were reasonably minor and were able to be fixed with little diminishment of the user experience. Across projects, researchers and project managers noted the importance of combining the photo capture with the audio/text narratives so as to gain a clearer understanding of the context and meaning behind the photos that were taken. Based on the ongoing feedback obtained from users, we have continued to enhance app usability and functions, particularly with respect to successfully engaging residents with low technology literacy and educational levels—for whom the app was originally developed [45]. Researchers and residents alike have noted how successful use of the app in capturing relevant local environment features can engender its own feelings of efficacy and empowerment, which in turn can help to set the stage for resident participation throughout the subsequent *Our Voice* steps.

In addition to the Discovery Tool app, other accompanying project support tools include password-protected access to a secure web platform for reviewing and processing Discovery Tool data; project-specific visual representation of all data collected by community members; a program implementation toolkit that includes community meeting facilitation guides, *Our Voice* technology user manuals, action planning templates, and advocacy training resources; a project-specific administrative dashboard that supports project coordination, tracking and documentation of project activities and outcomes, and formulation of project reports; and participation in the *Our Voice* Global Network, which fosters cross-project learnings and collaborations.

### 3.5. Maintaining Project Momentum to Achieve Successes and Address Challenges

Maintaining momentum throughout a project to achieve its goals requires a willingness of citizen scientists and community partners and facilitators to continue to engage over the time it takes to accomplish proposed changes. Sustaining this participation is challenging, given busy schedules and competing demands on people’s time. Also, a clear understanding of who is responsible for implementing solutions is important, to prevent misunderstandings. Strategies used by the projects described above include meeting in convenient, familiar settings, providing transportation to meetings, providing refreshments at meetings, being flexible about meeting attendance (i.e., not every participant will make every meeting), and identifying a smaller group of spokespeople who are willing and able to represent the larger citizen scientist group in meeting with stakeholders, presenting data, and advocating for specific changes.

Once the initial project period ends, continuing momentum is also desirable but may be challenging if involvement from original project facilitators ceases due to turnover or lack of funding. Participant-generated ideas for continuing the work long-term include transitioning the facilitation role to community groups with a shared interest or vision; raising awareness of age-friendly needs and solutions among business leaders and service providers; and spreading use of such citizen science methods to other local communities and groups. The lessons learned from the projects described underscore both the promise of using a participatory citizen science approach and the need for sustained engagement from program facilitators and residents alike in ensuring that the action steps generated come to fruition. In addition, the improvements in empowerment, collective efficacy, and social cohesion among older adults described in these projects [24,61] can potentially be harnessed to achieve further gains in promoting age-friendly community objectives.

## 4. Discussion

The suite of projects presented in this paper contribute to the expanding evidence base supporting the use of participatory research methods to advance global activities promoting age-friendly communities. Although it has been noted frequently that older adults represent an important resource and can make valuable contributions in this area, they are often among the last to be included in local decision-making activities [8,13]. The WHO’s Age-Friendly Cities initiative and other movements (e.g., the “village movement” in the United States) [72] have set the stage for an increased co-creation process between older residents and community stakeholders. In doing so, such initiatives have served as catalysts for shaping communities in ways that can increasingly support the world’s aging population. While different participatory research approaches have been used to foster older adult engagement in this area [14,27], involvement of older adults in actual citizen science partnerships with researchers has varied along a participation continuum [13,15,22]. This continuum includes, at one end, obtaining periodic input from older residents and, at the other end, actively partnering with them throughout the full scientific process—including problem identification, data collection, analysis, and interpretation, prioritization of issues, and co-development of relevant solutions [22]. The Manchester approach is one example of a “by the people” research initiative that combines top–down with such bottom–up strategies for engaging and empowering older adults and stakeholders alike in promoting age-friendly environments [14,27]. With mounting recognition of the limitations of “top–down” age-friendly policies in many regions of the world [14], further development of such bottom–up approaches is likely to be increasingly needed. The *Our Voice* citizen science initiative has sought to expand and extend older adult participation in this type of bottom–up, “by the people” approach through engaging residents in a systematic, research-to-action method. Among potential distinctions of this approach relative to others is the way in which it systematically leverages the power of emerging information and communication technology to enlighten and engage residents across different socioeconomic strata and cultures. From this technology-enabled data collection activity through data interpretation and real-world application, older adults become drivers, as opposed to bystanders, in co-creating more age-friendly environments.

The results thus far from the *Our Voice* approach support the promise of this method for promoting age-friendly neighborhoods and communities in varying cultures and circumstances. Changes associated with this method to date include improving built environment outdoor spaces and infrastructure that can promote neighborhood walkability and pedestrian safety; increasing access to a variety of physical activity opportunities; enhancing the usability of local parks; furthering social connections in a community to better enable respect and inclusion for all its members; increasing older adult mobility and comfort in a health care setting; and assuring that clear, timely and trustworthy communication and information is available to older adults so that they are able to more fully access the community and health services they need. Together, the projects described demonstrate how aging adults from diverse backgrounds and conditions can learn how to employ mobile technology to capture relevant barriers to and enablers of healthy living and aging. They can then learn how to successfully engage relevant stakeholders and service providers to compel meaningful yet realistic age-friendly changes in their local environments. A strength of this model has been its focus specifically on less-advantaged, ethnically diverse populations that often have not had a voice in their communities [24]. The importance of including traditionally marginalized groups has been noted to be a critical issue for the age-friendly movement to address [73].

Another potentially important way in which this method contributes to the age-friendly environments field is that it employs standardized, manualized and scalable intervention methods that, based on the suite of studies conducted thus far, appear able to have impacts across all of the different domains represented in the WHO age-friendly cities framework. The specific tools and methods available through this approach allow facilitating organizations to concretely move residents through the process of providing initial input through driving action in their communities.

### 4.1. Limitations

Among the limitations of the first-generation studies that have been conducted thus far are often small numbers of citizen scientists, although each study undertaken to date has resulted in some independently observed changes in built (e.g., physical infrastructure) and/or social environments (e.g., addition of age-friendly programs). With respect to numbers and selection of citizen scientists, it is important to note that while it is useful to describe who the citizen scientists are and how they were recruited in each study to advance implementation science in the field, the major outcomes of interest for this research are not at the individual level but at the environmental and policy levels. Thus, issues of selection bias are arguably of lesser impact than in scientific investigations focusing on participant outcomes at the individual level, where selection bias can have greater effects on the external validity of the outcomes in question. The primary role of citizen scientists, in contrast, is to be change agents who are not only involved in problem identification but are fully engaged in the intervention process itself. This point notwithstanding, impacts of the participatory intervention on citizen science behaviors and beliefs that can affect program success and resident responses (e.g., feelings of civic engagement, collective agency and efficacy, etc.) should be captured as additional variables of interest. Also, it remains important to select the types of communities and groups of citizen scientists that can particularly benefit from this type of research, in terms of health equity considerations. Such considerations have been a major driver underlying many *Our Voice* projects to date.

Additional limitations are the short follow-up periods, use of simple pre–post study designs, and varied evaluation methods. Second-generation studies in this area could advance the field through including larger and more diverse groups of older adults; and employing stronger designs, including comparison-group designs as well as natural experiments. Testing intergenerational citizen science programs could further our understanding of potential cross-generation synergies that could increase local impact. Studies that address a number of these issues are currently underway, including a US National Institutes of Health-funded (PHS #5R01CA211048) cluster-randomized clinical trial called Steps for Change, summarized in Table 1. This trial is evaluating the impacts of physical activity programming, with and without the addition of *Our Voice,* on individual and neighborhood physical activity levels in older adults living in or around senior affordable housing sites in northern California. In addition, a natural experiment is underway in Bogotá, Colombia which includes evaluation of the impacts of the *Our Voice* method in understanding the benefits and costs of a new cable car transit system for vulnerable populations living there. The study is using citizen science methods to identify, prioritize, and communicate the most salient negative and positive features impacting health and quality of life among groups of low-income residents living in the cable car system jurisdiction. It is also being used to facilitate a consensus and advocacy-building process among community members, policymakers, and researchers there.

### 4.2. Future Directions

In addition to the information that has been learned to date, there are a number of future directions in which this line of research can go to maximize its value and returns, including the following recommendations:

- Continue to expand the scientific rigor, methods, and designs commensurate with this type of community-enabled research. This includes quasi-experimental pre–post comparison group designs [49], as well as, when appropriate and feasible, experimental designs comparing the efficacy of health interventions with and without the addition of “by the people” citizen science methods.
- Measurement batteries also should be expanded to more thoroughly capture change at different levels of impact, including at the individual, interpersonal, environmental and policy levels [44]. In addition, greater cross-project harmonization of the measurement batteries being employed in each study would accelerate cross-project learnings [44].
- Employ formal applications of qualitative comparative analysis to identify sets of conditions that are necessary and sufficient for successful implementation of the *Our Voice* model. Among the potential factors that may have favorable or detrimental effects on successful implementation are the following [43]: citizen scientists’ perceptions of whether the changes made adequately address the problems they identified; whether the problems and solutions align or conflict with priorities of other local groups, including neighborhood groups, local governments, etc.; the extent to which identified decision makers and stakeholders have the authority, interest, and resources to accomplish the proposed changes; whether or not there are committed champions dedicated to supporting, promoting, and driving the changes; and the best methods for promoting sustained resident involvement to enhance the chances of ripple effects, that is, the spread of community-engaged citizen science activities to other issues.
- Test innovative approaches for capturing, over time, all of the varied impacts of such resident-engaged approaches—both intended and unexpected—through using systematic methods such as ripple effects mapping (REM) [74]. REM is a participatory qualitative methodology where participants and stakeholders visually map together the “snowballing” trajectory of project-related activities and outcomes that accrue over time [74,75]. To thoroughly capture such effects, which can occur beyond the formal end of a project, lengthening the duration of project assessment activities is recommended.
- Prospectively combine use of the WHO age-friendly checklist and *Our Voice* methods to evaluate age-friendly features and identify feasible barriers and solutions across all eight topic areas.
- Increase both the number and types of intergenerational citizen science projects to build better communication and understanding between and across generations, which could in turn accelerate community impacts [23,48].
- Expand the data capture capabilities of this platform through adding mobile sensors and other assessment tools to the Discovery Tool walks that are occurring around residents’ communities. In this manner, a more comprehensive picture of the potential health and quality of life impacts of specific community locales and features can emerge. An example of this is having residents use a wrist-worn sensor that collects electro-dermal and heart rate activity in helping to identify locations along a particular walking route that engender increases in arousal or stress [52].
- Explore linkages to other data platforms through introducing this type of complementary resident-centric, micro-environmental perspective to computational, epidemiological, and other “big data” scientists, given that these data are typically missing in “big data” sets. Such resident-collected data may be particularly relevant for vulnerable populations, including older adults [76].

## 5. Conclusions

The *Our Voice* Global Citizen Science Research Initiative and Network represent a promising approach to building age-friendly communities for older adults and other residents, irrespective of the circumstances, locations, or cultures in which people live. By combining contextually oriented data gathering methods that are part of participatory action research, while tapping into the skills, knowledge, and networks of older adults, *Our Voice* may help to accelerate the adoption and implementation of contextually relevant, feasible, and sustainable community change to foster healthy aging. These projects individually and collectively illustrate the observation, found in other *Our Voice* projects and emphasized by scientific thought leaders such as the anthropologist Margaret Mead, that small groups of committed residents working together can make a difference in their communities.

A longer-term goal of this global research initiative is to build an interactive world map of resident-collected data and project results along with other resources that can be shared by researchers, non-academic and government organizations, and residents alike. This type of collaborative undertaking can help to advance the vision laid out by the WHO and other organizations in building a true path to achieving global age-friendly communities along with health equity in under-resourced communities and beyond.

## Figures and Tables

**Table 1:** Examples of *Our Voice* older adult projects completed or in process.

Location and Project Focus	Description and Participants (N = Sample Size)	Community Features Identified		Strategies Proposed and Changes Enacted
		Positive	Negative	
**BUILT ENVIRONMENT**				
*Haifa, Israel*^1^ Age- and activity-friendly cities [1]	Ethnically and socioeconomically diverse adults ages 50 years and older (N = 59) from 4 neighborhoods in Haifa	Easy access to commercial and leisure facilities Attractive buildings Benches, public restrooms	Poor sidewalk condition Street stairs in disrepair Obstacles to sidewalk use Neglected lots Traffic noise, pollution	Identified safest routes to destinations Developed a senior-friendly “golden path” walking map Began to work with Mayor’s office and local organizations and businesses to initiate changes (e.g., improved aesthetics) to support walking
*East Palo Alto, CA (USA)* Senior-friendly activity and food environments [16,27]	Assessment and advocacy around food and physical activity environments of local neighborhoods (N = 12 ethnically diverse low-income older adults living in senior public housing)	A wide variety of good quality fruits and vegetables available in local stores	A street outside the housing setting with high pedestrian and vehicular traffic had no designated place to cross safely	Participants partnered with a local non-profit garden-based education organization, which provided education, gardening tools, and seeds to develop a community garden Sustained relationships between study participants and city officials, resulting in a more coherent focus on creating an age-friendly community Allocation of significant government dollars for built environment improvements and public health inclusion in the city’s general plan
*San Mateo County, CA (USA)* Food access and transportation [18]	Examination of the factors that facilitate or hinder access to food, and food-related behavior, followed by advocacy for positive environmental and policy-level changes. (N = 23 ethnically diverse, food insecure, low-income older adults)	Lower prices Access and availability of healthy food in the store Freshness and quality of produce	Price promotions for unhealthy food The presence of unhealthy food The price of items not being displayed within view or at all Higher prices Having to visit multiple stores for cheaper prices Poor personal health	Local organizations made information available in multiple languages about food assistance and transportation services At 3 months, 84% of study participants had either shared new information/resources, contacted a local decision or policy maker, and/or signed up for a new service (e.g., SNAP, shuttle service) At 6 months, a senior advocacy team (SAT) was formed and convened an open forum, presented concerns and solutions to city and county policymakers (N = 5); Within 4 days, improved street signage and curb painted red for better visibility SAT participated in the State Capital’s Fifth Annual Affordable Senior Housing Resident Advocacy Day in Sacramento, CA SAT partnered with an elementary school to address pedestrian and bicycle safety concerns due to high-speed traffic City Transportation and Planning Department installed a device to measure traffic and speed on the street, then later installed pedestrian flashing light signals and modified crosswalk for safety
*North Fair Oaks, CA (USA)* Neighborhood walkability and security across generations [25]	Assessment of neighborhood built-environment features that help or hinder physical activity (N = 10 low-income Latinx adults, mean age 71 years and 10 low-income Latinx adolescents, mean age 13 years)	Having attractive destinations and amenities to visit The aesthetic ‘feel’ of the neighborhood Good quality sidewalks	Trash Poor quality sidewalks Personal safety	Resident-informed Community Resource Guide was compiled Resident recommendations included the following: Trash: report illegal dumping, make signs asking people to clean up after pets, form volunteer groups to clean up trash, increase knowledge about trash pick-up days for larger items (e.g., furniture), request additional public trash bins from the city, require and enforce that apartment owners should supply residents with appropriate trash disposal facilities Personal safety: form a neighborhood watch association; replace graffiti with murals; work with the city to learn how to complete forms, start a petition, initiate action; increase police patrols, open the park and use cameras to monitor activity; increase security on the footbridge (patrols and cameras) Sidewalks: report unsafe sidewalks to Dept. of Public Works Residents worked with local media to highlight priority issues, and article about the project appeared in national media A steering committee of local municipal and service organizations was formed to address issue of illegal dumping and trash The County Manager’s office conducted research into best management practices on illegal dumping, engaged with other cities and counties around this issue, and has explored use of web and mobile technologies to allow resident reporting of trash
*Cuernavaca, Mexico* Supporting intergenerational active living across socioeconomic strata [19]	Testing the acceptability and feasibility of using the Our Voice approach to assess walkability environments in four neighborhoods in Mexico, stratified according to socioeconomic status and walkability. (N = 32 adults, 9 adolescents)	Presence of parks or recreational facilities Having destinations to visit	Poor sidewalk quality Presence of trash Negative street characteristics Unpleasant aesthetics (e.g., graffiti) Feeling unsafe Unleashed dogs Limited disabled access Lack of crosswalks Poor quality of parks and recreational facilities	Discussed creation of a neighborhood committee and campaign to encourage neighbors to use leashes and clean up after their dogs Adults and adolescents discussed acceptable forms of public art/graffiti together Neighborhood watch programs recommended to combat crime Strategies identified to promote increased social cohesion in the neighborhood
*Curitiba, Brazil* Neighborhood environmental characteristics and physical activity among older adults	Older adults from neighborhood areas with high and low walkability and SES (N = 32)	Presence and quality of sidewalks Land use mix (proximity of services, e.g., markets, bakery)	Functional characteristics walking surface/pattern and streets connectivity Aesthetics issues as bad designed and/or maintained streetscape and presence of physical disorder	Strategy development in process
*Santa Clara and San Mateo Counties, CA, (USA)* Improving walkability around affordable senior housing sites	Older adult residents and neighbors of affordable housing sites, enrolled in a physical activity intervention (N = 69)	Murals on electrical boxes Community Gardens Flashing light sidewalks Traffic signs Park and community centers within walking distance Clean amenities on walking routes	Cracked Sidewalks Overgrown Shrubs Lack of curb ramps Lifted manhole covers Narrow/No sidewalks Cars parked on sidewalks Walking time given to cross intersections Visibility of bus stop signs Trash or hazardous waste along walking paths	Residents wrote letters to describe safety concerns with sidewalk cracks and proposed that if they could not be repaired, they at least be marked with paint to make them visible to residents Emailed community center staff requesting that they relay their concerns about negative community features to the proper departments; Information was relayed to the Maintenance division Sidewalk cracks were repaired on a major avenue Thank-you letters were sent to volunteers at a nicely maintained rose garden At a local community center, gravel was added to level the ground between a walking track and sidewalk to prevent a walking hazard Dirt and overgrown shrubs on sidewalk were cleared out Sidewalk was repainted red to stop cars from parking A stop sign that had fallen was repaired Put up a new stop sign at a local park to make entry easier Put in a cross walk near one of the affordable housing sites Improved visibility of bus stops signs and phone numbers to call to obtain the bus schedule Painted sidewalk curve at local community center to prevent falls Cracked, uneven sidewalk repair at another community center
*Manitoba, Canada* Creating an age-friendly campus	Older people (≥65 years) assessed overall age-friendliness of the University of Manitoba’s Fort Garry campus (N = 10)	Fitness programming for older people (including walking paths and places to cycle) Libraries Restaurants Positive campus environment Positive customer service experiences	Several missing handrails, automatic door openers, bench seating along walkways Absent, confusing, or hard to read campus signage Unsafe walking surfaces (tripping hazard) Lack of separation between cyclists and pedestrian traffic Cost and availability of parking for older people with accessibility concerns	Comprehensive physical accessibility scan of campus to identify overlooked areas (completed as part of provincially-mandated legislation and ongoing accessibility audits of campus) Adding additional bench seating Increasing walkway maintenance and reconstruction budget Will vastly improve the quality and amount of signage to building entrances, pedestrian walkways, university roads, and parking lots (currently part of a larger wayfinding project on campus) Adding more pedestrian crossings and dedicated bike lanes Adding more short-term and accessible parking spaces
*Bath, Kent, Keynsham, Wolverhampton, UK* Increasing age- and activity-friendliness of diverse communities	Increasing the age and activity friendliness of geographically and socioeconomically diverse communities (N = 19 older adults, 66 ± 7 years old)	Sidewalk availability and dropped curbs Access to facilities including recreational facilities (museums, shops), daily destinations (parks, green spaces and benches) and public transport. Community spirit (i.e., friendly people, supportive networks, community hubs) Variety of local amenities Signposting of walking/cycling routes	Damaged sidewalks Obstacles on sidewalks (e.g., leaves, trash bins) Aesthetics: Graffiti, unkept gardens, overgrown trees/bushes, flower beds, vandalism Neighborhood safety: lack of signs and lighting, high traffic volume Public crossing characteristics (i.e., long distances between crossings, insufficient crossing duration) Personal Safety: groups of young people, stray dogs Accessibility and Walkability: unreliable public transport, challenges walking on cobbled streets, limited access to parks, shops, benches Air pollution	Citizen scientists articulated the following goals and strategies: Provide accommodations for people with compromised walking abilities or who use walking aids Provide unobstructed access to good quality and safe sidewalks Provide sheltered benches that accommodate different abilities Provide local amenities for coffee, sociability Provide public toilets Advertise the walking/cycling routes Subsidize active forms of travel Enhance roads to reduce traffic volume Put neighborhood watch schemes in place Provide more trash bins to reduce litter Park patrols to help older adults feel safer Provide communal picnic areas to give more of a safe and communal feeling Restrict big lorries to use only bigger roads and motorways
*Temuco, Chile* Neighborhood environmental characteristics that promote quality of life and physical activity among older adults	Community-dwelling older adults from neighborhoods with different socioeconomic status and walkability (N = 60, ≥60 years)	Availability and proximity of services, goods Availability of green spaces, sidewalks Government-funded programs to improve neighborhoods Bus stop renovations and new signage Participatory decisions for improving common spaces (public art)	Sidewalks need maintenance Some street corners need better signs and measures to reduce vehicle speed Illegal garbage disposal in some corners People selling drugs in some areas Lack of support to maintain surveillance cameras under operation	Strategy development in process Several stakeholders have been identified for the implementation of potential solutions such as the Council program for older adults, Regional Secretary of Transport, Council Department of Transport, Regional Secretary of Housing and Urbanism, Regional Secretary of Aging, Police
*East San Jose, CA (USA)* Intergenerational approaches to building a healthy community	Collaboration with SOMOS Mayfair organization, and local Public Health Department; (N = 50 multi-aged residents	Public Art	Low access/utilization of public spaces for physical activity (PA) Not enough public art Lack of affordable housing Abandonment and dangerous infrastructure	Presented findings to Mayor and City Council Memorandum of understanding (MOU) with School District to allow access to a local soccer field Development of Scavenger Hunt cards to attract local park use Creation and dissemination of “Walking Loop” cards through new partnership with California Walks and resident walking groups New PA programming
**SOCIAL ENVIRONMENT**				
*Anchorage, Alaska*^1^ Safe and healthy aging for older LGBT residents	Analysis of environmental factors that impact feelings of social isolation (N = 8)	LGBT community advocacy organization Natural beauty of Alaska	Limited safe public transportation options Treacherous winter walk/drive conditions Lack of LGBT-welcoming venues Fear for personal safety based on historical discrimination	LGBT elder-friendly events, social opportunities, and meetings held at Anchorage Senior Center, local cafes, and other venues Increased ridesharing coordination to American Association of Retired Persons (AARP) or SAGE events Offer of new educational events with Anchorage Senior Center, business leaders and senior service providers
*Cijin, Taiwan*^1^ Senior-friendly places for social and recreational activities	Older adults with mean age 70 years (SD = 10), 33% women, all with a high school education (N = 15)	Some aesthetics	No places to socialize Abandoned buildings Dysfunctional sewers Broken sidewalks Personal safety issues from motorbikes	Prioritized abandoned buildings and personal safety as particular high-priority issues An abandoned building was identified to turn into a community center where older adults could safely gather and socialized
**COMMUNITY AND HEALTH SERVICES**				
*Brisbane, Australia*^1^ Ensuring a mobility-friendly geriatric medical rehabilitation unit	Older adults in a medical rehabilitation unit (N = 10; 8 confined to wheelchairs)	A community garden and coffee shop at rehab unit Windows providing views of the sky and some greenery	Swinging vs. sliding doors Hard-to-reach cupboards Drab décor Steeply inclined entryway Bed curtains provided little privacy	Moved a patient kitchenette and drinking fountain to more accessible locations Changing curtains to allow for greater privacy and which brightened décor Re-arranged furniture to allow greater wheelchair navigation Lowered paper towel dispensers in bedrooms for easier access

Note. ^1^ Project results described in further detail below.

**Table 2:** Descriptive information on implementation of Israel’s *Our Voice* projects in five additional cities.

City	Neighborhood	City Description	Local Partnering Organizations	Citizen Scientist Population (N = Sample Size)	Partnership and Recruitment Process	Our Voice Facilitation
Lod	Sharett	In total, 74,000 residents In total, 72.5% Jewish and 27.5% Arab In total, ~33% new immigrants from former Soviet Union and Ethiopia	Municipal Welfare Department JDC Eshel JDC Ashalim Liaisons from the “Better Together” program for community work with older adults	N = 30 Participants in a digital literacy course and other club activities Primarily women over age 68	Outreach to working to engage older adults Identification of “good fit” opportunities (i.e., digital literacy course and Better Together program) Development of joint work agreement Approval from City Welfare Department	Organized by the Better Together project liaison together with representatives from the *OV* project and the older adult club Two meetings for each group, to introduce the project and train the participants Facilitators accompanied citizen scientists on Discovery Tool (DT) walks as needed/appropriate
	Ganei Aviv			N = 15 Russian-speaking immigrants in digital literacy course and/or other club programs		
Tel Aviv	Shapira	In total, 8500 residents Primarily low socioeconomic status High population of foreign workers living alongside old-time residents	Municipal Welfare Department Clubs for older adults	N = 25 Participants in physical activity groups at a club for older adults Neighborhood activists (non-club members) Equal numbers men/women Most aged 70 or above Some with physical impairments (e.g., using walkers)	Recruitment through “home groups” to maximize comfort	*Our Voice* project lead coordinated via local club liaison and community social worker Engaged younger volunteers as guides to accompany participants, help alleviate technology anxieties, and answer questions Three community meetings to introduce program, recruit, and train on use of DT Created local WhatsApp groups to ensure successful use of the DT and data upload
	Mo’adon Mitchell	Old neighborhood with long-term residents, many post WWII immigrants Generally high socioeconomic status	The Mitchell Center for older adults, which offers diverse activities and serves as a social center for its members Municipal Welfare Department	N = 9 Over 70 years of age Eight women and one man	Recruitment by a national service volunteer at the club Outreach to those comfortable with using mobile devices Offered tutorials and support to others Individualized orientation to *OV* project and DT	Regular consultation and supervision between *OV* lead and local project facilitators Two meetings offering DT instruction and thematic analysis of DT data collected National volunteer service and community social worker reg-ularly contacted participants
	Hatikva	In cluster of three neighborhoods with ~20,000 residents Most foreign-born In total, 10%–15% older adults In total, 33% on welfare	Municipal Welfare Department Clubs for older adults	N = 14 Mainly Sephardi In total, 12 women and two men aged 65 and above	The municipality’s community work team selected the neighborhood and engaged the local social worker The social worker recruited participants through the club and among resident activists	Community social worker facilitated process with support of national *OV* program liaison Social worker and two volunteers personally accompanied participants on DT walks Three community meetings to introduce program, recruit, and train on use of DT
	Ajami	Old neighborhood with narrow, crowded streets Mix of Arabs and Jews	Municipal Welfare Department Clubs for older adults	N = 35 Arab women aged 65–70	Municipality community work team selected the neighborhood club because many women already active Club director, social worker and program liaison led recruitment Recruitment lasted a month	Club director and social worker joined residents on DT walks Ongoing consultation with *OV* national liaison Two meetings to introduce project, recruit, and select themes Plan to present the findings to the relevant municipality officials
Bat Yam	Gordon	High proportion of immigrants from former Soviet Union Ranked 14th in population and 55th in geographic size The third most crowded city in Israel	JDC “Better Together” program Local Community Center	N = 10 Club members/retirees already active in the club In total, seven women, three men	Open invitation to all interested club members Presentation and DT training by the program liaison and the club director	Club director led process together with the program liaison Daily contact and consultation Joined residents on DT walks Two meetings for recruitment, DT training, and theme selection Presentation of findings and proposed solutions to municipal officials
	Negba		Negba Community Center (part of the Community Center company)	N = 10 Women aged 75+ Most already active in club and low SES	The club liaison recruited, and the program liaison trained for DT use	
Petah Tikvah	Menachem Ratzon	Over 244,000 residents (fifth most populous in Israel) The population growth rate is 1.6% annually	Municipal Welfare and Health Clubs for older adults	N = 12 Women aged 75+ Generally already “active and concerned”	Recruitment by club director Two-week recruitment period Participants selected based on enthusiasm and willingness to volunteer	Co-facilitated by club director and club’s national service volunteer Facilitators accompanied participants on DT walks Two meetings Recruitment and training Theme selection Awaiting meeting with municipal officials
	Sela			N = 8 Women		
	Beit Dani			N = 8 In total, 7 women, 1 man, 70+		
	Smilansky			N = 8 In total, five women, three men, 70+		
Jerusalem	Beit Hakerem	High socioeconomic status Relatively homogeneous population of secular native Israelis	JDC Eshel “Community for Generations” program City of Jerusalem Community Welfare department Local branch of the scout movement	N = 38 In total, 23 older adults (15 women and eight men) In total, 15 high school student members of the Scout movement	The Community for Generations director recruited participants Reached out to the Scout movement for an intergenerational connection Recruitment lasted ~two months	Led by Community for Generations director with help of Scouts’ Community Involvement liaisons (high school students) Sessions initially separated by group, then joint sessions with retirees and students Collaboration with Scouts extended process to 6 months DT walks intergenerational; decided together what to document Aim of building shared vision for the neighborhood, for all ages
	Har Homa	Mainly young families Approximately 28,000 residents Some 1800 older adults	Jerusalem municipality	In total, 15 women aged 68 and above	Recruitment lasted ~two months and included an initial session to introduce the program Those interested joined a second session to learn how to use the DT	Led by club director and program liaison Direct contact with retirees and accompanied them on DT walks Two meetings to introduce project, recruit, and select themes Presentation to officials pending

## Supplementary Materials

The following are available online at https://www.mdpi.com/1660-4601/17/5/1541/s1. Video S1: Our Voice: Citizen Science for Health Equity https://www.youtube.com/watch?v=sYcYXh51Bl0.

## References

[1] Luepker R.V., Yi Z., Crimmins E.M., Carrière Y., Robine J.M. (2006). Increasing longevity: Causes, consequences, and prospects. Longer Life and Healthy Aging, International Studies in Population.

[2] World Health Organization (2016). Global Strategy and Action Plan on Ageing and Health (2016–2020).

[3] Beard J.R., Officer A., De Carvalho I.A., Sadana R., Pot A.M., Michel J.P., Lloyd-Sherlock P., Epping-Jordan J.E., Peeters G.G., Mahanani W.R. (2016). The World report on ageing and health: A policy framework for healthy ageing. Lancet.

[4] Angel R.J., Angel J.L., Hill T.D. (2015). Longer lives, sicker lives? Increased longevity and extended disability among Mexican-origin elders. J. Gerontol. B Psychol. Sci. Soc. Sci..

[5] Subramanian S.V., Kubzansky L., Berkman L., Fay M., Kawachi I. (2006). Neighborhood effects on the self-rated health of elders: Uncovering the relative importance of structural and service-related neighborhood environments. J. Gerontol. B Psychol. Sci. Soc. Sci..

[6] Pruchno R.A., Wilson-Genderson M., Cartwright F.P. (2012). The texture of neighborhoods and disability among older adults. J. Gerontol. B Psychol. Sci. Soc. Sci..

[7] Gibney S., Zhang M., Brennan C. (2019). Age-friendly environments and psychosocial wellbeing: A study of older urban residents in Ireland. Aging Ment. Health.

[8] Buffel T., Phillipson C., Scharf T. (2012). Ageing in urban environments: Developing “age-friendly” cities. Crit. Soc. Policy.

[9] Austin C.D., Camp E.D., Flux D., McClelland R.W., Sieppert J. (2005). Community development with older adults in their neighborhoods: The elder friendly communities program. Fam. Soc..

[10] Novek S., Menec V.H. (2014). Older adults’ perceptions of age-friendly communities in Canada: A photovoice study. Ageing Soc..

[11] Lui C.W., Everingham J.A., Warburton J., Cuthill M., Bartlett H. (2009). What makes a community age-friendly: A review of international literature. Australas. J. Ageing.

[12] Menec V.H., Means R., Keating N., Parkhurst G., Eales J. (2011). Conceptualizing age-friendly communities. Can. J. Aging.

[13] Corrado A.M., Benjamin-Thomas T.E., McGrath C., Hand C., Laliberte Rudman D. (2019). Participatory Action Research With Older Adults: A Critical Interpretive Synthesis. Gerontologist.

[14] Buffel T., Phillipson C. (2018). A Manifesto for the Age-Friendly Movement: Developing a New Urban Agenda. J. Aging Soc. Policy.

[15] English P.B., Richardson M.J., Garzon-Galvis C. (2018). From Crowdsourcing to Extreme Citizen Science: Participatory Research for Environmental Health. Annu. Rev. Public Health.

[16] Gottwald S., Laatikainen T.E., Kyttä M. (2016). Exploring the usability of PPGIS among older adults: Challenges and opportunities. Int. J. Geogr. Inf. Sci..

[17] Jankowski P. (2009). Towards participatory geographic information systems for community-based environmental decision making. J. Environ. Manag..

[18] Brown G., Kyttä M. (2014). Key issues and research priorities for public participation GIS (PPGIS): A synthesis based on empirical research. Appl. Geogr..

[19] Zolkafli A., Liu Y., Brown G. (2017). Bridging the knowledge divide between public and experts using PGIS for land use planning in Malaysia. Appl. Geogr..

[20] Kahila-Tani M., Kytta M., Geertman S. (2019). Does mapping improve public participation? Exploring the pros and cons of using public participation GIS in urban planning practices. Landsc. Urban Plan..

[21] Ives C.D., Oke C., Hehir A., Gordon A., Wang Y., Bekessy S.A. (2017). Capturing residents’ values for urban green space: Mapping, analysis and guidance for practice. Landsc. Urban Plan..

[22] King A.C., Winter S.J., Chrisinger B.W., Hua J., Banchoff A.W. (2019). Maximizing the promise of citizen science to advance health and prevent disease. Prev. Med..

[23] Winter S.J., Rosas L.G., Romero P.P., Sheats J.L., Buman M.P., Baker C., King A.C. (2015). Using citizen scientists to gather, analyze, and disseminate information about neighborhood features that affect active living. J. Immigr. Minority Health.

[24] King A.C., Winter S.J., Sheats J.L., Rosas L.G., Buman M.P., Salvo D., Rodriguez N.M., Seguin R.A., Moran M., Garber R. (2016). Leveraging citizen science and information technology for population physical activity promotion. Transl. J. ACSM.

[25] Sheats J.L., Winter S.J., Romero P.P., King A.C. (2017). FEAST: Empowering community residents to use technology to assess and advocate for healthy food environments. J. Urban Health.

[26] Garon S., Paris M., Beaulieu M., Veil A., Laliberte A. (2014). Collaborative partnership in age-friendly cities: Two case studies from Quebec, Canada. J. Aging Soc. Policy.

[27] Buffel T., McGarry P., Phillipson C., De Donder L., Dury S., De Witte N., Smetcoren A.S., Verté D., Sánchez-González D., Rodríguez-Rodríguez V. (2016). Developing age-friendly cities: Case studies from Brussels and Manchester and implications for policy and practice. Environmental Gerontology in Europe and Latin America: Policies and Perspectives on Environment and Aging.

[28] Buffel T., Skyrme J., Phillipson C., Shek D., Hollister R. (2017). Connecting research with social responsibility: Developing age-friendly communities in Manchester, UK. University Social Responsibility and Quality of Life: Concepts and Experiences in the Global World.

[29] Rowbotham S., McKinnon M., Leach J., Lamberts R., Hawe P. (2017). Does citizen science have the capacity to transform population health science?. Crit. Public Health.

[30] National Academies of Sciences, Engineering and Medicine, Health and Medicine Division, Board on Population Health and Public Health Practice, Committee on Community-Based Solutions to Promote Health Equity in the United States (2017). Communities in Action: Pathways to Health Equity.

[31] Kelty C., Panofsky A. (2014). Disentangling public participation in science and biomedicine. Genome Med..

[32] Silverton J. (2009). A new dawn for citizen science. Trends Ecol. Evol..

[33] World Health Organization (2007). Global Age-Friendly Cities: A Guide.

[34] World Health Organization (2007). Checklist of Essential Features of Age-Friendly Cities.

[35] Rappaport J. (1984). Studies in empowerment: Introduction to the issue. Prev. Hum. Serv..

[36] General Household Survey (2002). People’s Perceptions of Their Neighborhood and Community Involvement: Results from the Social Capital Module of the General Household Survey 2000.

[37] Sallis J.F., Owen N., Glanz K., Rimer B.K., Lewis F.M. (2002). Ecological models of health behavior. Health Behavior and Health Education: Theory, Research, and Practice.

[38] King A.C. (2015). Theory’s role in shaping behavioral health research for population health. Int. J. Behav. Nutr. Phys. Act..

[39] Bandura A. (2006). Toward a psychology of human agency. Psychol. Sci..

[40] Ryan R.M., Deci E.L. (2000). Self-determination theory and the facilitation of intrinsic motivation, social development, and well-being. Am. Psychol..

[41] Rothman J., Rothman J., Tropman J.E. (2001). Approaches to community intervention. Strategies of Community Intervention.

[42] Coleman J. (1988). Social capital in the creation of human capital. Am. J. Sociol..

[43] Damschroder L.J., Aron D.C., Keith R.E., Kirsh S.R., Alexander J.A., Lowery J.C. (2009). Fostering implementation of health services research findings into practice: A consolidated framework for advancing implementation science. Implement. Sci..

[44] Hinckson E., Schneider M., Winter S.J., Stone E., Puhan M., Stathi A., Porter M.M., Gardiner P.A., Dos Santos D.L., Wolff A. (2017). Citizen science applied to building healthier community environments: Advancing the field through shared construct and measurement development. Int. J. Behav. Nutr. Phys. Act..

[45] Buman M.P., Winter S.J., Sheats J.L., Hekler E.B., Otten J.J., Grieco L.A., King A.C. (2013). The Stanford Healthy Neighborhood Discovery Tool: A computerized tool to assess active living environments. Am. J. Prev. Med..

[46] Buman M.P., Winter S.J., Baker C., Hekler E.B., Otten J.J., King A.C. (2012). Neighborhood Eating and Activity Advocacy Teams (NEAAT): Engaging older adults in policy activities to improve food and physical environments. Transl. Behav. Med..

[47] Seguin R.A., Morgan E.H., Connor L.M., Garner J.A., King A.C., Sheats J.L., Winter S.J., Buman M.P. (2015). Rural food and physical activity assessment using an electronic tablet-based application, New York, 2013–2014. Prev. Chronic Dis..

[48] Rosas L.G., Salvo D., Winter S.J., Cortes D., Rivera J., Rodriguez N.M., King A.C. (2016). Harnessing Technology and Citizen Science to Support Neighborhoods that Promote Active Living in Mexico. J. Urban Health.

[49] Rodriguez N.M., Arce A., Kawaguchi A., Hua J., Broderick B., Winter S.J., King A.C. (2019). Enhancing Safe Routes to School Programs through Community-Engaged Citizen Science: Two Pilot Investigations in Lower Density Areas of Santa Clara County, California, USA. BMC Public Health.

[50] Moran M., Werner P., Doron I., HaGani N., Benvenisti Y., King A.C., Winter S.J., Sheats J.L., Garber R., Motro H. (2015). Detecting inequalities in healthy and age-friendly environments: Examining the Stanford Healthy Neighborhood Discovery Tool in Israel. International Research Workshop on Inequalities in Health Promoting Environments: Physical Activity and Diet.

[51] Chrisinger B.W., Ramos A., Shaykis F., Martinez T., Banchoff A.W., Winter S.J., King A.C. (2018). Leveraging citizen science for healthier food environments: A pilot study to evaluate corner stores in Camden, New Jersey. Front. Public Health.

[52] Chrisinger B., King A.C. (2018). Stress experiences in neighborhood and social environments (SENSE): A pilot study to integrate the quantified self with citizen science to improve the built environment and health. Int. J. Health Geogr..

[53] Tuckett A.G., Freeman A., Hetherington S., Gardiner P.A., King A.C., on behalf of Burnie Brae Citizen Scientists (2018). Older adults using Our Voice Citizen Science to create change in their neighborhood environment. Int. J. Environ. Res. Public Health.

[54] Moran M.R., Werner P., Doron I., HaGani N., Benvenisti Y., King A.C., Winter S.J., Sheats J.L., Garber R., Motro H. (2016). Exploring the objective and perceived environmental attributes of older adults’ neighborhood walking routes: A mixed methods analysis. J. Aging Phys. Act..

[55] Moran M., Van Cauwenberg J., Hercky-Linnewiel R., Cerin E., Deforche B., Plaut P. (2014). Understanding the relationships between the physical environment and physical activity in older adults: A systematic review of qualitative studies. Int. J. Behav. Nutr. Phys. Act..

[56] Plough A.L. (2015). Measuring What Matters: Introducing a New Action Framework.

[57] King A.C., Sallis J.F., Frank L.D., Saelens B.E., Cain K., Conway T.L., Chapman J.E., Ahn D.K., Kerr J. (2011). Aging in neighborhoods differing in walkability and income: Associations with physical activity and obesity in older adults. Soc. Sci. Med..

[58] Physical Activity Guidelines Advisory Committee (2018). 2018 Physical Activity Guidelines Advisory Committee Scientific Report.

[59] Moran M., Werner P., Doron L., Benvenisti Y., HaGani N., King A.C., Winter S.J., Sheats J. (2015). Health Promoting Environments: Participatory Action Research for Health and Age-Friendly Neighbourhoods (Research Report).

[60] McKenzie T.L., Cohen D.A. (2006). SOPARC (System for Observing Play and Recreation in Communities)—Description and Procedures Manual.

[61] Winter S.J., Buman M.P., Sheats J.L., Hekler E.B., Otten J.J., Baker C., Cohen D., Butler B.A., King A.C. (2014). Harnessing the potential of older adults to measure and modify their environments: Long-term successes of the Neighborhood Eating and Activity Advocacy Team (NEAAT) Study. Transl. Behav. Med..

[62] Kemperman A., van den Berg P., Weijs-Perrée M., Uijtdewillegen K. (2019). Loneliness of older adults: Social network and the living environment. Int. J. Environ. Res. Public Health.

[63] Marmot M. (2005). Social determinants of health inequalities. Lancet.

[64] Lin Y.Y., Huang C.S. (2016). Aging in Taiwan: Building a Society for Active Aging and Aging in Place. Gerontologist.

[65] Chou Y., Hua J., Banchoff A.W., Winter S.J., Liou D., King A.C. (2018). Harnessing technology and citizen science to support age-friendly neighborhoods in Taiwan. Ann. Behav. Med..

[66] Gonzales G., Henning-Smith C. (2017). Health Disparities by Sexual Orientation: Results and Implications from the Behavioral Risk Factor Surveillance System. J. Community Health.

[67] King D.K., Holdorf M., Sudbeck D., Schmidt J.P. (2019). Safe and Healthy Aging for LGBT Elders Using Citizen Science: Discoveries from “Our Voice SAGE Alaska”.

[68] Sampson R.J., Raudenbush S.W., Earls F. (1997). Neighborhoods and violent crime: A multilevel study of collective efficacy. Science.

[69] de Jong Gierveld J., Van Tilburg T. (2006). A 6-item scale for overall, emotional, and social loneliness: Confirmatory tests on survey data. Res. Aging.

[70] Yang J., Chu Y., Salmon M.A. (2018). Predicting Perceived Isolation among Midlife and Older LGBT Adults: The Role of Welcoming Aging Service Providers. Gerontologist.

[71] Brooks-Cleator L.A., Giles A.R., Flaherty M. (2019). Community-level factors that contribute to First Nations and Inuit older adults feeling supported to age well in a Canadian city. J. Aging Stud..

[72] Greenfield E.A., Oberlink M., Scharlach A.E., Neal M.B., Stafford P.B. (2015). Age-friendly community initiatives: Conceptual issues and key questions. Gerontologist.

[73] Gonyea J.G., Hudson R.B. (2015). Emerging models of age-friendly communities: A. framework for understanding inclusion. Public Policy Aging Rep..

[74] Washburn L.T., Traywick L., Thornton L., Vincent J., Brown T. (2018). Using Ripple Effects Mapping to evaluate a community-based health program: Perspectives of program implementers. Health Promot. Pract..

[75] Welborn R., Downey L., Dyk P.H., Monroe P.A., Tayler-Mackey C., Worthy S.L. (2016). Turning the tide on poverty: Documenting impacts through ripple effect mapping. Community Dev..

[76] Satariano W.A. (2006). The Epidemiology of Aging: An Ecological Approach.

